# Lipidomic identification of plasma lipids associated with pain behaviour and pathology in a mouse model of osteoarthritis

**DOI:** 10.1007/s11306-020-01652-8

**Published:** 2020-02-27

**Authors:** P. Pousinis, P. R. W. Gowler, J. J. Burston, C. A. Ortori, V. Chapman, D. A. Barrett

**Affiliations:** 1 Centre for Analytical Bioscience, Advanced Materials and Healthcare Technology Division, School of Pharmacy, University of Nottingham, Nottingham, UK; 2 Pain Centre Versus Arthritis, University of Nottingham, Nottingham, UK; 3 School of Life Sciences, University of Nottingham, Nottingham, UK

**Keywords:** Osteoarthritis, Pain, LC–MS lipidomics, Destabilisation of the medial meniscus

## Abstract

**Introduction:**

Osteoarthritis (OA) is the most common form of joint disease, causing pain and disability. Previous studies have demonstrated the role of lipid mediators in OA pathogenesis.

**Objectives:**

To explore potential alterations in the plasma lipidomic profile in an established mouse model of OA, with a view to identification of potential biomarkers of pain and/or pathology.

**Methods:**

Pain behaviour was assessed following destabilisation of the medial meniscus (DMM) model of OA (n = 8 mice) and compared to sham controls (n = 7). Plasma and knee joints were collected at 16 weeks post-surgery. Plasma samples were analysed using ultra-high performance liquid chromatography accurate mass high resolution mass spectrometry (UHPLC-HR-MS) to identify potential differences in the lipidome, using multivariate and univariate statistical analyses. Correlations between pain behaviour, joint pathology and levels of lipids were investigated.

**Results:**

24 lipids, predominantly from the lipid classes of cholesterol esters (CE), fatty acids (FA), phosphatidylcholines (PC), *N*-acylethanolamines (NAE) and sphingomyelins (SM), were differentially expressed in DMM plasma compared to sham plasma. Six of these lipids which were increased in the DMM model were identified as CE(18:2), CE(20:4), CE(22:6), PC(18:0/18:2), PC(38:7) and SM(d34:1). CEs were positively correlated with pain behaviour and all six lipid species were positively correlated with cartilage damage. Pathways shown to be involved in altered lipid homeostasis in OA were steroid biosynthesis and sphingolipid metabolism.

**Conclusion:**

We identify plasma lipid species associated with pain and/or pathology in a DMM model of OA.

**Electronic supplementary material:**

The online version of this article (10.1007/s11306-020-01652-8) contains supplementary material, which is available to authorized users.

## Introduction

Osteoarthritis (OA) is the most common form of joint disease, characterized by pain and disability (Felson et al. 2000; Hinman and Crossley 2007). Recent estimates suggest that the global burden of knee OA affects approximately 250 million people. In USA alone, approximately 21 million people present with OA associated symptoms, while it is estimated that the number of individuals is set to double by 2020 (Hunter et al. 2014). Currently there are no disease modifying drugs for OA and treatment of the associated pain is hampered by lack of efficacy and/or unwanted side-effects of treatments. Total joint replacement remains the most effective treatment for OA pain (Lui et al. 2015). It is increasingly recognised that the slow progression of OA pathology, coupled with the poor relationship between radiographic OA and the phenotype and magnitude of pain experienced, limits treatment strategies to prevent the development of chronic pain. Biochemical markers (in blood, urine or synovial fluid) of OA have the potential to reflect changes in joint structure to monitor disease progression (Lotz et al. 2013) and OA pain (Valdes et al. 2017, 2018). There is a pressing clinical need for biomarkers of OA that detect the disease in its early stages, where progression of disease may still be responsive to pharmacotherapy. Such biomarkers could also contribute to personalized medicine and the identification of new drug targets (Mobasheri and Henrotin 2015).

The role of a broad spectrum of lipid mediators, including fatty acids, sphingolipids, and eicosanoids in cartilage degradation in OA is increasingly recognised (Masuko et al. 2009). Lipid dysregulation in all compartments of the joint is considered an important feature of this disease (Courties et al. 2015; Sun et al. 2016). Lipidomics, which can be defined as the system-wide characterization of lipids and their interaction with other biochemicals and cells (Spener 2003), has dramatically advanced over the past decade, due to developments in analytical technologies, such as mass spectrometry (MS) and chromatography, reviewed in (Hyotylainen and Oresic 2016).

Previous studies highlight the value of a lipidomic approach to identify potential biomarkers of OA pain and pathology. For example, a global lipidomic analysis of human synovial fluid identified changes in 66 phospholipid species in early OA versus late OA (Kosinska et al. 2013) and changes in the synovial fluid levels of 21 sphingolipids in early OA compared to late OA (Kosinska et al. 2014). Comparison of the synovial fluid lipidomes of human OA versus canine OA identified similarities in the lipid composition between the two species, in particular for early stages of OA (Kosinska et al. 2016). However, collection of synovial fluid is invasive and there are limitations in obtaining synovial fluid samples from control healthy volunteers. The utility of plasma to search for biomarkers of OA progression has been highlighted by the first untargeted (global) lipidomics study of human OA plasma (Castro-Perez et al. 2010), which revealed an altered lipid metabolism associated with OA.

Animal models of OA have advanced understanding of the mechanisms of pain and facilitate studies of the complex relationship between pathology and pain behaviour (Little and Zaki 2012). Destabilization of the medial meniscus (DMM) in the mouse (Glasson et al. 2007) is a slowly progressing surgical model of OA, which mimics key features of the clinical pathology and pain responses. There are few lipidomic studies in animal models of OA and these have been focused on targeted lipid (oxylipin) analysis. In a model of inflammatory arthritis, He et al. (He et al. 2015) reported differences in oxylipin plasma levels in collagen induced arthritis (CIA) mice compared to control mice using a targeted LC–MS/MS oxylipin method with both univariate and multivariate analysis. Previously we used a novel targeted oxylipin LC–MS/MS method to measure oxylipin and endocannabinoid profiles in different tissues from the rat monosodium iodoacetate (MIA) model of OA pain, compared to control rats (Wong et al. 2014).

The aim of the present study was to use untargeted lipidomics LC–MS analysis in conjunction with multivariate and univariate analysis to identify potential changes in plasma lipidome in the DMM model of OA, to identify related pathways of lipid metabolism altered in OA, and to investigate potential correlations with pain behaviour and joint pathology.

## Material and methods

### Reagents and materials

A Milli-Q water purification system (Millipore, MA, USA) was used in the preparation of deionized water (18.2 MΩ). Acetonitrile and chloroform were HPLC grade purchased from Fischer Scientific (Loughborough, UK). Methanol (LC–MS grade) and ammonium acetate were purchased from Sigma-Aldrich (Dorset, UK). Isopropanol (LC–MS grade) was obtained from Fischer Scientific (Loughborough, UK).

### Animals

Experiments were performed on male C57BL/6 mice (Charles River, Margate, Kent, UK) aged 8–9 weeks at start of surgery in accordance with the UK Animal (Scientific Procedures) Act (1986). All procedures were approved by the University of Nottingham Ethical Review Committee and IASP guidelines. All animals were group-housed in a temperature-controlled environment (22 ± 1 °C) and maintained on a 12-h light/dark cycle with access to an identical diet and water ad libitum. A total of 15 male C57BL/6 mice (n = 7 in sham control group, n = 8 in DMM group) were used for this study.

### Induction of DMM model

The surgery to generate the DMM model of OA was performed under brief isoflurane anaesthesia. An incision was made over the medial meniscus; a blunt dissection was then used to open the knee joint capsule and the mediomeniscotibial ligament (MMTL) was transected to destabilise the medial meniscus (Glasson et al. 2007). Sham operated mice underwent the same procedure except for the transection of the MMTL.

### Pain behaviour testing

Changes in weight distribution (weight bearing, WB) between the left (ipsilateral) and right (contralateral) knees were assessed using an incapacitance meter (Linton Instruments UK) as previously described (Bove et al. 2003). Hindpaw withdrawal thresholds (PWT) to mechanical stimulation of the hindpaw were measured using calibrated von Frey monofilaments using the up down method, as previously described (Sagar et al. 2010). The timeline of behavioural pain measurements is provided in Electronic Supplementary Information (ESI, Fig. S1A). The time-course of changes in WB and PWT in DMM mice is provided in Figs. S1B, C.

### Joint pathology

The ipsilateral knee joints were fixed in 10% neutral buffered formalin post-mortem before being decalcified in a 10% ethylenediaminetetraacetic acid (EDTA) solution for 10 days. Coronal sections (5 µm thickness) were stained with haematoxylin and eosin before chondropathy in the medial tibial plateau was scored according to the OARSI scoring system (Glasson et al. 2010), (Fig. S2).

### Plasma collection and lipid extraction

On post-operative (PO) day 112, following the last set of pain behaviour tests, mice were euthanized. Blood was collected, centrifuged at 15,871×*g* for 5 min, at room temperature, and the plasma frozen immediately in liquid nitrogen. Note the volume of blood (1 mL) required for the global lipidomic analysis prevented a longitudinal study of the potential changes in lipidomics over the time-course of the study. For this reason we focused on the late time point (week 16) when pain behaviour had been significant for a number of weeks and joint pathology was known to be significant as well. All samples were stored at − 80 °C until lipidomic extraction (Folch et al. 1957) (see ESI) and LC–MS analysis.

### Liquid chromatography–mass spectrometry lipidomic analysis

LC–MS analysis was performed based on a method previously published from our group (Haoula et al. 2015). Briefly, chromatographic separations were performed on an ACE 2 C18 HPLC column (20 × 2.1 mm, 2 μm particle size; Aberdeen, UK), maintained at a temperature of 40 °C and a flow rate of 600 µL/min. Mobile phases consisted of (A) 60:40 acetonitrile:10 mM aqueous ammonium acetate and (B) 90:10 isopropanol:10 mM ammonium acetate in acetonitrile. A binary gradient from 32 to 97% B was used with a total run time of 4 min. The injection volume was 10 µL and was the same as the mobile phase A composition.

Mass spectrometry was performed on an Orbitrap Exactive MS (ThermoFisher Scientific, Hemel Hempstead, UK) acquiring data simultaneously in full scan ion mode (*m/z* 100–1200, resolution 50,000 at *m/z* 200) in positive and negative ionisation modes. The capillary voltage was maintained at 25 V in the positive ion mode and at 27 V in the negative ion mode. The voltages of tube lens and skimmer in positive mode were set to 115 and 22 V respectively. Negative mode voltages of tube lens and skimmer were set to 140 and 28 V respectively. The flow rates of sheath gas, auxiliary gas and sweep gas for both positive negative modes were adjusted to 30, 15 and 5 (arbitrary units). The capillary temperature and heater temperature were maintained at 350 and 300 °C respectively in both positive and negative modes.

### Lipidomics data analysis

Raw UHPLC-HR-MS data from the DMM and control mice samples were acquired using Xcalibur v2.1 software (Thermo Scientific, Hemel Hempstead UK). The full datasets from DMM group and control group were imported and pre-processed in SIEVE (Version 2.1, Thermo Fisher Scientific Inc., USA) using normalisation to total ion intensity (TIC). Ions imported for further analysis into SIMCA-14 (Umetrics, Umea, Sweden) were included as follows: (a) ions with non-zero peak areas with an RSD of peak areas less than 30% in QCs and (b) ions that have an RSD of peak areas less than 30% in each group (control and OA) (Gika et al. 2016; Vorkas et al. 2015).

The processed datasets analysed by principal component analysis (PCA) and orthogonal projections to latent structures discriminant analysis (OPLS-DA) (Trygg et al. 2007) using pareto (Par) scaling. VIP (Variable Importance in the Projection) and p(corr) were used (V-Plot) to find statistically changed ions (Chang et al. 2017). Ions with VIP > 1.5 and p(corr) > |0.4| were subjected to univariate analysis with False Discovery Rate (FDR) at 5% level for correction of multiple comparisons using Prism v.7 (Graph Pad, San Diego, California, USA). The performance of the analytical method was validated by monitoring a representative set of plasma lipids in pooled quality control (QC) samples, injected throughout the LC–MS (following full equilibration) run to assess RSD(%) of retention time (RT) shifts and peak areas.

Tentative identification of significant lipids was achieved by using accurate mass determinations (up to 5 ppm mass accuracy) to search appropriate metabolite databases: LIPID MAPS (www.lipidmaps.org), the Human Metabolome database (www.hmdb.ca) and METLIN (https://metlin.scripps.edu). Statistically significant lipid species were confirmed by MS/MS fragmentation experiments in Q-Exactive (Thermo, UK) using LipidSearch (Breitkopf et al. 2017, Peake et al. 2013) software v4.1 (Thermo Fisher Scientific, CA, USA) (see ESI for more information).

Metabolite identification confidence was classified using identification levels as proposed previously (Sumner et al. 2007). Putative statistically significant lipids between sham and DMM groups were categorized according to their VIP score. Furthermore, in order to validate the differentially expressed lipids the sensitivity, specificity and the area under the receiver-operating characteristic (ROC) curve for binary classification of the OPLS-DA models were also calculated from the respective Monte-Carlo cross validation prediction (www.metaboanalyst.ca).

In addition, based on the identified biomarkers, the plasma lipidomic pathway analysis was performed using Metabolic Pathway Analysis (MetPA), a tool in MetaboAnalyst (www.metaboanalyst.ca), to reveal the most relevant pathways related to OA. The impact value of these pathways calculated from pathway topology analysis above 0.1 was considered as the potential target pathway.

### Statistical analysis

Statistical analysis was performed using Prism v.7 (Graph Pad, San Diego, California, USA). Data are presented as mean ± SEM or box-and-whisker plots. Changes in weight bearing asymmetry and log transformed paw withdrawal thresholds (PWT) in DMM and sham operated mice over time were analyzed by two-way ANOVA with Bonferroni corrected multiple comparisons. Differences in medial tibial plateau cartilage damage between DMM and sham mice were analyzed by Mann–Whitney U test. Differences in lipid metabolite expression between DMM and sham mice were analyzed by two-tailed Welch’s t-test. Correlations between pain behavior (weight bearing asymmetry and log PWT at the final behavioural timepoint) and MTP cartilage damage with the expression of lipid metabolites were analyzed by Pearson’s Correlation Coefficient. A value of p < 0.05 was considered significant for all analyses.

## Results

### Pain behavior and joint pathology of DMM model

Changes in weight-bearing (WB) were evident in DMM mice, with a significant decrease in WB on the injured side from day 56 post-DMM surgery, compared with sham controls. Changes in WB remained stable until the end of the study (Fig. S1A). Similarly, ipsilateral hindpaw withdrawal thresholds were lowered from day 49 post DMM surgery until the end of the study (Fig. S1B). There were significant differences in pain behavior between the DMM and sham control group for the later stages of the study. At the end of the study (day 112) DMM mice had a significant MTP score indicative of cartilage damage, compared to the sham controls (Fig. S2).

### Lipidomic profiling using UHPLC-HR-MS and identification of potential biomarkers

The lipidomics analysis met accepted quality criteria with close clustering of the plasma QCs in PCA, and with RSD% of peak areas from selected lipids and RT being less than 15% and 2%, respectively (ESI, Table S1).

**Fig. 1:**
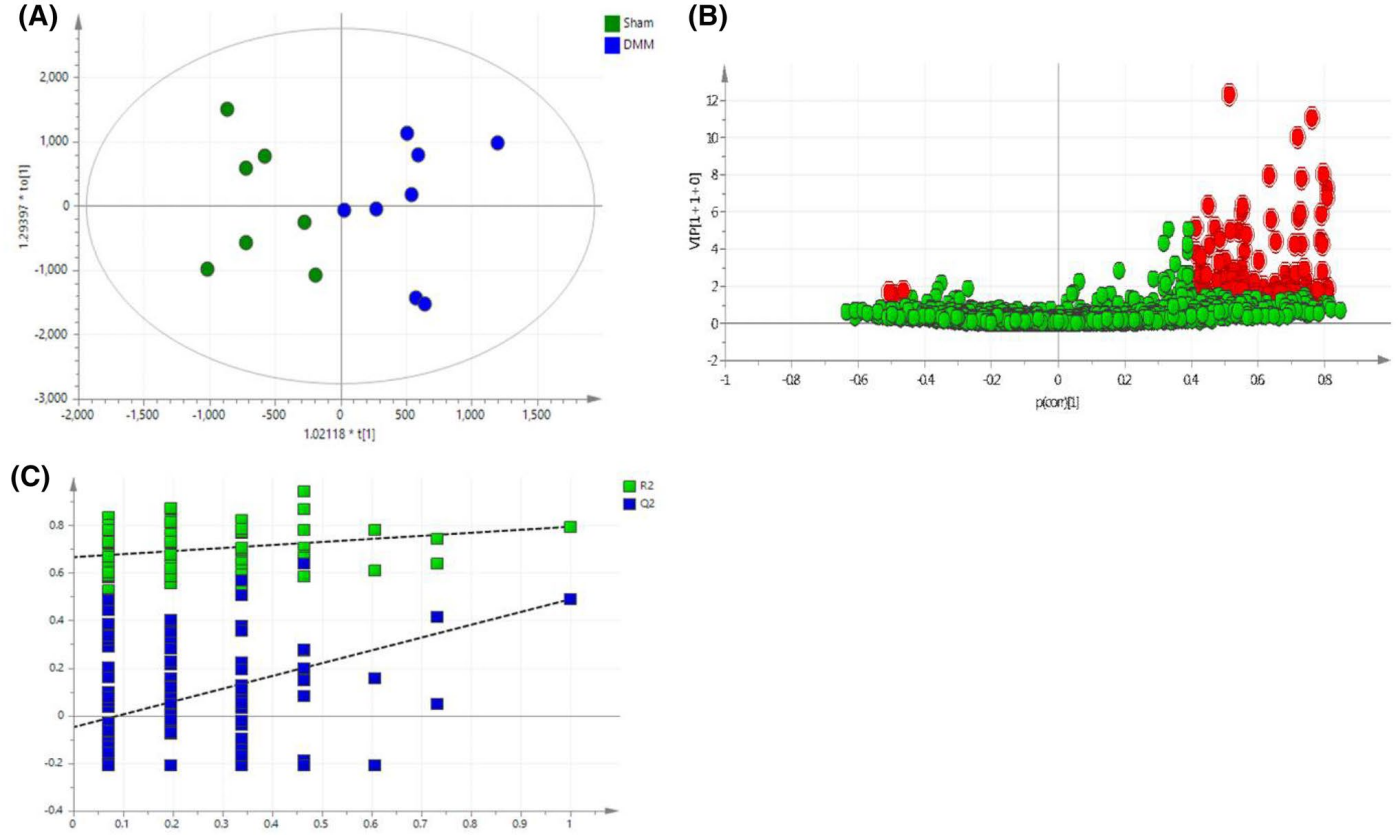
Multivariate analysis of global lipidomics in the DMM mouse model. **a** OPLS-DA score plot of plasma from DMM (n = 8, blue) and, sham mice (n = 7, green). The score plots show a good separation between DMM and sham mice (R2X = 0.547, R2Y = 0.794, and Q2 = 0.407). **b** V-plot with p(corr) and VIP values. Features with VIP > 1.5 and p(corr) >|0.4| are highlighted in red. **c** A permutation test performed with 100 random permutations on generated PLS-DA model; R2 is the explained variance, and Q2 is the predictive ability of the model. Low value of Q2-intercept depicts the high predictability of the model

**Fig. 2:**
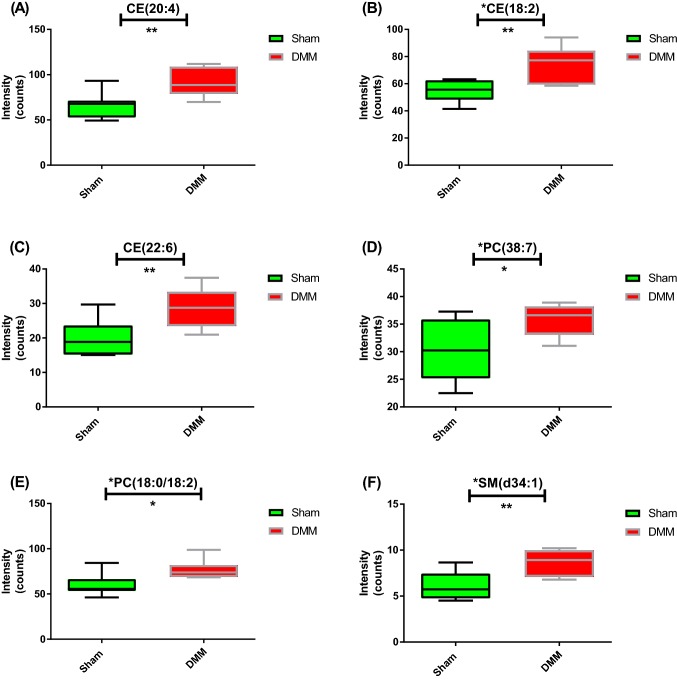
Lipids discriminating DMM samples (red) from sham samples (green). Box-and-whisker plots illustrating levels differences for the six lipid biomarkers between DMM and sham groups of mice. Welch’s t-test was applied. *p < 0.05; **p < 0.01

An unsupervised PCA was initially performed on the normalized data to identify trends in groups, clusters and potential outliers (ESI, Fig. S3). A supervised OPLS-DA model was generated (Fig. 1a) where R2X = 0.547, R2Y = 0.794, and Q2 = 0.407 showing good prediction of the model (Q2 > 0.4). The set of lipids that made significant contributions to the difference between the DMM and sham mice were identified using the V-Plot (Fig. 1b). The OPLS-DA permutation plot had low (negative) value of Q2-intercept confirming validation of the original model (Fig. 1c). Univariate statistical analysis using FDR (at level of 5%) revealed 24 differentially expressed lipids that were tentatively identified using metabolomics and lipidomics databases (METLIN, LIPIDMAPS, HMDB, and KEGG) (Table 1). For confirmation of lipid species, MS/MS fragmentation experiments (with LipidSearch software) using a pooled QC sample (ESI, Figs. S4–9) confirmed the identities of six of the 24 significant lipids and were assigned as CE(18:2), CE(20:4), CE(22:6), PC(18:0/18:2), PC(38:7), and SM(d34:1). The remaining lipids were assigned to class of lipid and are reported as a specific *m/z* ion, with a tentative identification (Table 1). LipidSearch outputs for the six identified lipids are given in ESI (Figs. S4–9). These six potential lipid biomarkers belong to three different lipid classes; sterols (STs), phospholipids (PLs), and sphingolipids (SLs). The tentatively identified lipids belong to the three aforementioned classes in addition to the class of fatty acids (FAs). Plasma levels of CE(20:4), CE(18:2), CE(22:6), PC(38:7), PC(18:0/18:2) and SM(d34:1) were significantly higher in the DMM group, compared to the sham group (Fig. 2).

**Table 1:** Identification of the 24 significant lipids (top 18 are shown) in DMM plasma (n = 7 and 8 mice/group, for sham and DMM, respectively) using UHPLC-HR-MS global lipidomics platform and OPLS-DA analysis

*m/z*	MS mode	Time (min)	VIP	Fold change^a^	Trend^b^	p value^c^	Putative lipid biomarker^d^	Confidence level^e^	Lipid class
318.240	POS	0.32	11.0	1.27	↑	0.00749	NAE 16:2	3	Fatty acyl
296.258	POS	0.28	10.0	1.49	↑	0.00731	FA(18:3)	3	Fatty acid
369.351	POS	2.86	8.0	1.39	↑	0.00598	Cholesterol	3	Sterol lipid
282.279	POS	0.73	7.9	1.39	↑	0.00893	LCB 18:2;1	3	Sphingolipid
690.619	POS	2.81	7.2	1.37	↑	0.00656	**CE(20:4)**	2	Sterol lipid
666.618	POS	2.88	6.7	1.38	↑	0.00368	**CE(18:2)**	2	Sterol lipid
844.609	NEG	2.15	5.5	1.26	↑	0.0164	**PC(18:0/18:2)**	2	PC
613.492	POS	0.32	4.4	1.44	↑	0.00376	CE(14:3)	3	Sterol lipid
806.569	POS	1.45	4.4	1.17	↑	0.0129	PC(38:6)	3	PC
714.619	POS	2.77	4.2	1.43	↑	0.00841	**CE(22:6)**	2	Sterol lipid
804.551	POS	1.53	3.3	1.19	↑	0.0190	**PC(38:7)**	2	PC
473.401	NEG	1.37	2.8	1.07	↑	0.0130	Sitosterol	3	Sterol lipid
256.268	POS	0.6	2.7	1.24	↑	0.00797	LCB 16:1;1	3	Sphingolipid
316.321	POS	0.79	2.6	1.13	↑	0.0217	FA(19:0)	3	Fatty acid
700.628	POS	2.72	2.3	1.43	↑	0.0114	Cer 43:2;3	3	Sphingolipid
703.575	POS	1.53	2.2	1.39	↑	0.00680	**SM(d34:1)**	2	Sphingolipid
290.209	POS	0.27	1.8	1.31	↑	0.00866	NAE 14:2	3	Fatty acyl
326.378	POS	0.66	1.8	1.45	↑	0.00334	FA(22:1)	3	Fatty acid

Lipids are sorted by their VIP scores (descending). Online databases METLIN, KEGG, HMDB and LIPIDMAPS were used to assign masses (*m/z*) to putative lipid species. Mass accuracy is considered less than 5 ppm. Fold change between DMM vs sham groups is shown. p values generated after applying FDR correction are given. Lipids identified by MS/MS experiments in Q Exactive with LipidSearch software) are highlighted in bold, when available. Lipid classes are also given

^a^DMM/sham group ratio (mean values from each group were used)

^b^DMM vs sham groups

^c^p value adjusted after FDR correction (5%) applied (GraphPrism v.6)

^d^Lipid abbreviations: *NAEN*-acylethanolamine, *FA* fatty acid, *LCB* long-chain bases, *CE* cholesteryl ester, *PC* phosphocholine, *Cer* ceramide, *SM* sphingomyelin

### Prediction and diagnostic performance test

**Fig. 3:**
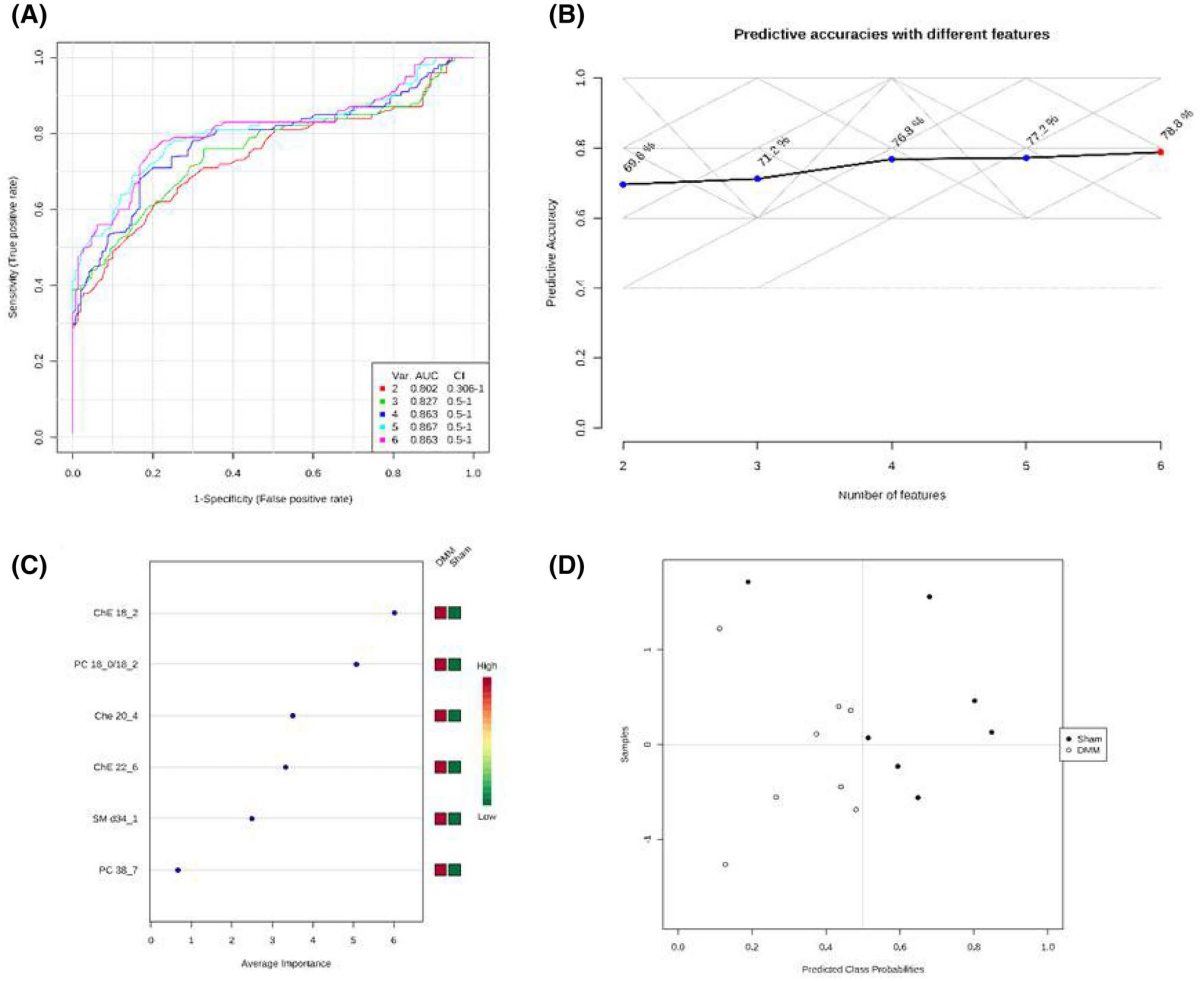
Comparison of different variables based on ROC curves **a** the legend shows the feature numbers and the AUCs of the five models, **b** the predictive accuracies with different features based on ROC curves, **c** the average importance of six lipids based on ROC curves, in descending order of importance, and **d** prediction of DMM and sham mice using MCCV analysis. The prediction of the model depends on the area under the curve (AUC) provided by ROC analysis: the greater the AUC, the better the prediction of the model

ROC analysis was performed to validate the OPLS-DA analysis and test the applicability of the six identified lipid biomarkers in separating DMM mice and sham control. Figure 3a shows a group of ROC curves for models established by using different lipids selected by the filter approach. Five models were generated. The top two lipids [CE(18:2) and PC(18:0/18:2)] were used to build classification model 1; the AUC value was 0.802 and the 95% confidence interval (CI) was 0.306–1. When all six lipids were used the AUC value was 0.863, while sensitivity was 100%, specificity of 85.7% (Fig. 3d), and predictive accuracy of 78.8% (Fig. 3b). On the basis of the selected biomarkers, ROC analysis revealed that the OPLS-DA model identified lipid biomarkers, sorted by their importance (Fig. 3c), that account for the differences between control and DMM mice.

### Metabolic pathway analysis

**Fig. 4:**
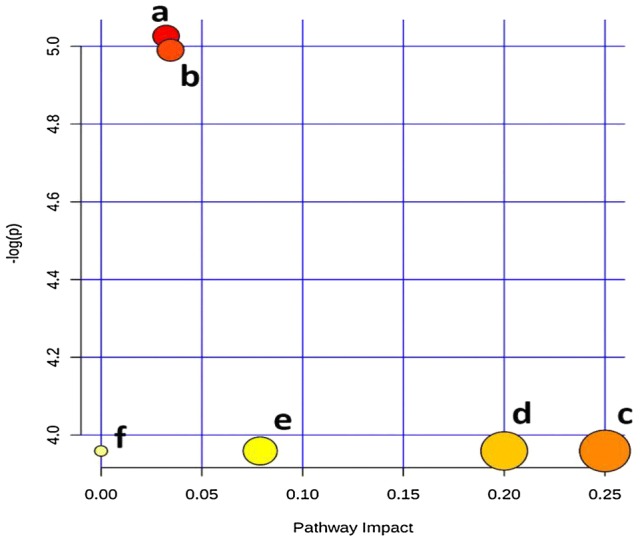
Summary of pathway analysis with MetPA: (a) steroid biosynthesis, (b) sphingolipid metabolism, (c) linoleic acid metabolism, (d) alpha-linolenic acid metabolism, (e) glycerophospholipid metabolism, and (f) arachidonic acid metabolism. The pathways depicted are listed from (a) to (f) in a descending order of importance, based on a combination of both the p values (y-axis) and impact (x-axis), according to Metabolic Pathway Analysis (MetPA) carried out in Metaboanalyst

The six lipids identified as potential biomarkers of OA were related to steroid (i.e. cholesterol) biosynthesis (CEs), sphingolipid metabolism [SM(d34:1)], linoleic acid, alpha-linolenic acid, glycerophospholipid, and arachidonic acid metabolism [PC(18:0/18:2) and PC(38:7)] (Fig. 4). The pathway with the smallest p value (p < 0.05) is considered the metabolic route most significantly altered in the DMM OA model. Notably, p values (raw p values) for all pathways generated from Metaboanalyst were p < 0.05. For more details see (ESI, Table S2).

### Correlation analysis of lipid levels with pain behaviour and joint pathology

**Fig. 5:**
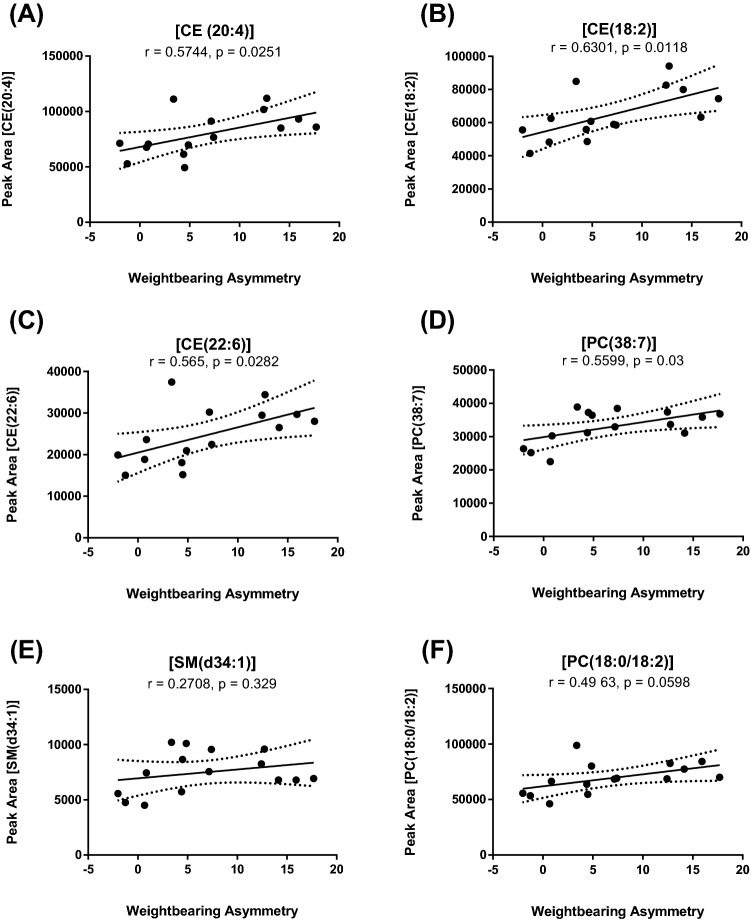
Correlation between levels of lipid metabolites and weight bearing asymmetry (WB) 16 weeks post DMM/sham surgery. Data analysed by Pearson’s Correlation Co-efficient. A positive correlation for all lipid biomarkers was observed. P and r values are shown

**Fig. 6:**
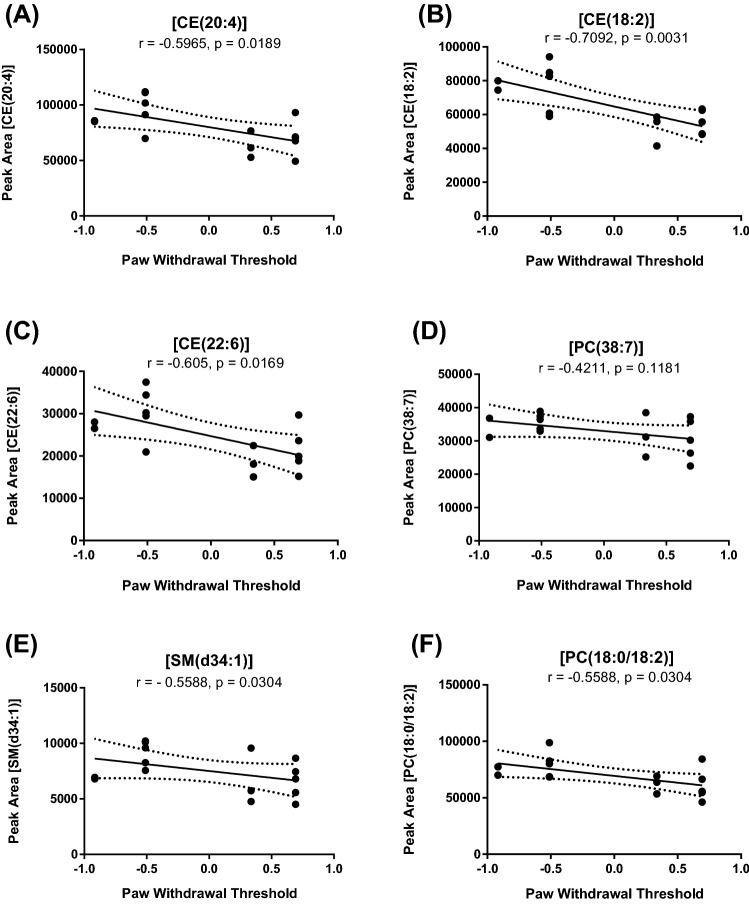
Correlation between levels of lipid metabolites and log transformed ipsilateral hindpaw withdrawal thresholds 16 weeks post DMM/sham surgery. Data analysed by Pearson’s Correlation Co-efficient. A negative correlation for all lipid biomarkers was observed. P and r values are shown

Pain on loading (weight-bearing asymmetry) and cartilage damage were not correlated with each other (ESI, Fig. S10). A Pearson correlation analysis evaluated potential associations of the six plasma lipids and pain behaviour parameters (WB and PWT). Plasma levels of CE(18:2), CE(20:4), CE(22:6), and PC(38:7) were positively correlated with changes in WB (p < 0.05, Fig. 5). There was a similar trend for PC(18:0/18:2) and SM(d34:1) (Fig. 5). There was a significant negative correlation between PWTs and plasma levels of CE(18:2), CE(20:4), CE(22:6), and PC(18:0/18:2) (Fig. 6). There was a similar trend for PC(38:7) and SM(d34:1) (Fig. 6). It is important to note that an increase in WB is indicative of pain behaviour, whereas a lowering of PWT is also indicative of pain behaviour. Overall CE(18:2), CE(20:4), and CE(22:6) levels were significantly correlated with both features of pain behaviour, suggesting that these plasma lipids may be biomarkers of OA pain behaviour. Lastly, all of the lipids with the exception of PC(38:7) were positively and significantly correlated with cartilage damage (Fig. S11).

## Discussion

### Lipid biomarkers of potential biological significance

The present study identified six potential plasma lipid biomarkers in the DMM model of OA pain, which were related to steroid biosynthesis (three CEs), sphingolipid metabolism [SM(d34:1)], linoleic acid, alpha-linolenic acid, glycerophospholipid, and arachidonic acid metabolism [PC(18:0/18:2) and PC(38:7)]. Although the fold changes of individual lipid levels between the sham and DMM mice were small (less than twofold), combining these six lipids together provided a valid model of OA pathology and pain as indicated by the ROC analysis (Fig. 3). Our data are supported by reports that the metabolic pathways underpinning the generation of these six lipids play important roles in OA pathogenesis, reviewed in (Masuko et al. 2009).

Sphingolipids (SLs) are a class of lipids that include ceramide (Cer) species, sphingomyelins (SMs) and more complex glycosphingolipids known to be present in SF (Lahiri and Futerman 2007). SLs are structural components of plasma membranes and bioactive molecules that regulate many processes including growth and differentiation, cellular signal transduction, and apoptosis in cells such as fibroblast-like synoviocytes (FLSs) (Baker et al. 2011; Patwardhan et al. 2016). Changes in sphingolipid metabolism have been associated with joint damage in a model of OA and selective inhibition of sphingosine kinase-2, a key enzyme in the sphingolipid pathway, attenuated histological damage and pain behaviour associated with MIA-induced OA in rats (Fitzpatrick et al. 2011). Our report that SM(d34:1) is a potential plasma lipid biomarker of OA is in agreement with a report by Kosinska et al. (2014) that the levels of 19 SMs, including SM(d34:1), were approximately twofold higher concentrations in SF from late OA versus early OA in humans. Furthermore, levels of 6 SMs were statistically increased by 1.7 fold in SF in a sub-group of OA sufferers (Zhang et al. 2014). Consistencies between the changes in the lipidome in plasma and SF in OA support further investigation of SLs as potential of plasma biomarkers.

Phospholipids (PLs) are essential components of all biological membranes and they contribute to boundary lubrication that is provided by SF (Kosinska et al. 2015). Our demonstration that plasma levels of PC(18:0/18:2) and PC(38:7) are significantly higher in the DMM model of OA are consistent with previous clinical data (Kosinska et al. 2013) showing alteration of phospholipid species between early OA and late OA SF in humans. The group of Kosinska (Kosinska et al. 2013) suggests that the phospholipid composition in SF is associated with increased friction, inflammation, and cartilage damage, ultimately reflecting the severity of human OA. A separate study demonstrated that levels of 24 glycerophospholipids in SF were significantly higher in patients in a subgroup of OA sufferers, with this effect more prominent for PC species (Zhang et al. 2014).

Amongst the significant lipids that were found to separate sham from DMM mice were NAE (14:2) and NAE (16:2). These lipids belong to the class of *N*-acylethanolamines (NAEs), bioactive lipids involved in many physiological processes including pain and inflammation (Bottemanne et al. 2018; Tsuboi et al. 2018).

Linoleic acid (18:2), an omega-6 fatty acid, which is a constituent part of two of our identified lipid biomarkers CE(18:2) and PC(18:0/18:2), is known to be metabolised via lipoxygenase, cyclooxygenase and cytochrome P450 (CYP) enzymes to a range of hydroxy- and oxo- metabolites that exhibit anti-inflammatory and immunomodulatory properties in OA (Chabane et al. 2009). Relevant to this observation, our group has recently reported that omega-6 FAs are increased in OA joints and downstream products of linoleic acid are associated with radiographic progression of OA (Valdes et al. 2018). The steroid biosynthesis pathway, identified in our study, has an important role in joint homeostasis, reviewed here (Farnaghi et al. 2017a, b), with both cartilage and bone severely affected by steroid hormone glucocorticoids, which are frequently used to treat inflammatory diseases.

Although identities were not confirmed for the lipids in the class of fatty acids (FAs), this family of lipids has established roles in OA (Sekar et al. 2017; Thomas et al. 2018). Recent studies support an involvement of n-3 poly-unsaturated fatty acids (PUFAs) and their anti-inflammatory and pro-resolving derivatives in OA. These lipids were identified in the OA joint, and were found to have beneficial effects on cartilage health in vitro and reduced pain in human OA and animal models (Mehler et al. 2016; Van de Vyver et al. 2018), reviewed here (Ioan-Facsinay and Kloppenburg 2018).

### Correlation analysis of lipids with pain and histology parameters

Potential relationships between levels of plasma lipid biomarkers and pain parameters (WB and PWT) at 16 weeks post-surgery were determined. Levels of the six plasma lipids identified as being elevated in the DMM model were significantly associated with pain behavior in the model of OA at this time-point. These data strengthen the validity of these six plasma lipids as potential biomarkers for established OA pain and are in agreement with the demonstration that increased levels of cholesterol transport molecules are associated with inflammation and pain in animal models of OA (Ioan-Facsinay and Kloppenburg 2018). However, it is still unknown whether these plasma lipids are related to pain during the onset and development of pain over time. Simvastatin, a lipid-lowering agent that blocks the production of cholesterol, inhibited mechanical hyperalgesia in a model of neuropathic pain, as well as attenuating the development of morphine tolerance (Vieira et al. 2017). In our study, plasma levels of the three CEs, PC(18:0/18:2), and SM(d34:1) which were significantly elevated in the DMM model, were correlated with the extent of articular cartilage damage in the knee joint at 16 weeks post-surgery. These data are consistent with evidence that a cholesterol-rich diet increased cartilage damage in a collagenase mouse model of OA (de Munter et al. 2013). In addition, mice fed a high-cholesterol diet had greater breakdown of the cartilage matrix compared to controls (Farnaghi et al. 2017a, b) and that the severity of diet-induced OA changes were attenuated by treatment with atorvastatin, a statin. In our study, both groups of mice followed the same diet and subsequently had the same cholesterol intake, hence any changes in cholesteryl esters (CEs) levels could be due to changes in cholesterol ester metabolism in the OA mouse model. More research into the role of CEs in OA pathophysiology is needed to further support our findings. Lastly, levels of both phospholipids (McDougall et al. 2017) and sphingomyelins (Kosinska et al. 2013) were correlated with cartilage damage in the MIA model of OA in the rat and in human OA patients, respectively.

## Conclusions

This is the first study using global LC–MS based lipidomics analysis in the established DMM mouse model of OA. Our LC–MS analysis of the whole lipidome in the plasma indicated differences in the profiles of 24 lipids between DMM and sham mice. Evidence is presented that a panel of six fully identified lipid species consisting of CEs, SMs and PCs may play roles in the pathogenesis of OA like chondropathy in this model. The six potential lipid biomarkers were related to different biochemical pathways, mainly steroid biosynthesis, sphingolipid and glycerophospholipid metabolism, consistent with previous studies. Lastly, correlation analysis suggests that the three CEs identified here are strongly associated with pain behavior and cartilage damage in the DMM model. While the findings need to be confirmed in large clinical studies in human knee OA, the identification of these six lipid biomarkers in small rodent studies in a non-invasive biological source, such as plasma, will help to unravel the pathogenesis and develop targeted therapies for OA.

## Electronic supplementary material

Below is the link to the electronic supplementary material.

Supplementary file1 (DOCX 1630 kb)
