# Supplementary material for: Lipidomic identification of plasma lipids associated with pain behaviour and pathology in a mouse model of osteoarthritis
Source: Metabolomics. 2020 Feb 27;16(3):32. doi: 10.1007/s11306-020-01652-8 (PMC7046574; doi:10.1007/s11306-020-01652-8)
Supplement: Supplementary file 1 — Supplementary file1 (DOCX 1630 kb) [file 11306_2020_1652_MOESM1_ESM.docx]

**Electronic Supplementary Material**

**Lipidomic identification of plasma lipids associated with pain behaviour and pathology in a mouse model of osteoarthritis**

Pousinis P^1^, Gowler PRW^2,3^, Burston JJ^2,3^, Ortori CA^1^, Chapman V^2,3^, & Barrett DA^1^

1 Centre for Analytical Bioscience, Advanced Materials and Healthcare Technology Division, School of Pharmacy, University of Nottingham, Nottingham, United Kingdom.

2 Pain Centre Versus Arthritis, University of Nottingham, Nottingham, United Kingdom.

3 School of Life Sciences, University of Nottingham, Nottingham, United Kingdom.

Corresponding Author: Victoria Chapman; Phone: 0115 82 30136, Fax: 0115 82 30142, Email: [Victoria.chapman@nottingham.ac.uk](mailto:Victoria.chapman@nottingham.ac.uk)

**Supplementary Methods**

**Lipid Extraction from Mouse Plasma**

Lipid extraction from rodent plasma for global lipidomics analysis (the whole lipidome) was performed using a modified method of the standard chloroform/methanol liquid-liquid extraction (LLE) ([Folch, Lees & Sloane Stanley, 1957](#_ENREF_2)) previously published from our group ([Haoula et al., 2015](#_ENREF_3)). Briefly, plasma samples were allowed to thaw (in ice-cold water bath) then were vortexed for 10 s. 50 μL of each plasma sample were added into pre-labeled polypropylene micro centrifuge tubes, 500 μL of ice-cold chloroform/methanol (1:2) were added into the micro centrifuge tubes, followed by gentle shaking (1,400 rpm for 10 min). After vortex-mixing (10s), 500 μL of MQ water was added, and the shaking/vortex procedures were repeated. Next, tubes were centrifuged at 15,871xg for 10 min at 4 °C. After centrifugation, the lower lipophilic and upper hydrophilic phases, which were divided by a protein layer, were separated by removing the upper aqueous phase with a pipette. Then, 100 μL of the lower lipophilic phase of each sample were removed into labeled poly-propylene centrifuge tubes, where an equal volume of isopropanol was added and vortexed. Lastly, samples were transferred into dark brown glass autosampler vials and stored at -80 °C, until LC-MS analysis.

**Lipid Identification using LipidSearch**

Lipids were identified using LipidSearch ([Breitkopf et al., 2017](#_ENREF_1); [Peake, 2013](#_ENREF_5)) software v4.1 (Thermo Fisher Scientific, CA, USA). LipidSearch v4.1 contains 18 lipid species and more than 1,500,000 theoretical MS/MS fragment ions in the database. Lipid identification is based on MS/MS match ([Yamada, Uchikata, Sakamoto, Yokoi, Fukusaki & Bamba, 2013](#_ENREF_8)). Mass tolerance for precursor (MS1) and fragment (MS2) was set to 5 ppm and 10 ppm respectively. The m-score threshold was set to 5 ([Xu, Wang, Jiao & Liu, 2018](#_ENREF_7)). The m-score measures the fit between experimental and expected product ion spectra and is based on the number of matching fragments; a higher m-score means higher identification reliability.

Furthermore, grades A, B, C, and D were all used for ID quality filter. Although a detailed description of A, B, C and D grades is given eloquently in a recent publication ([Breitkopf et al., 2017](#_ENREF_1)) a brief explanation is presented here. The ID quality A–B type is based on the identification of all features including head group, glycerol backbone and both fatty acid chains. The C–D type identifies the lipid class based on head group and backbone but may be lacking information in the MS2 spectra for one of the fatty acid chains or give information for the merged fatty acid chain. LipidSearch with A, B and C graded lipid identifications only were accepted for further analyses for this study. Also, all lipid classes in database including 66 subclasses were chosen for identification. Lastly, adducts of +H, +NH4 were used for positive mode search and –H, +CH3COO were selected for negative mode since ammonium acetate was used in mobile phases. The application of LipidSearch has been used in several studies ([Koelmel et al., 2019](#_ENREF_4); [Wali et al., 2016](#_ENREF_6)).


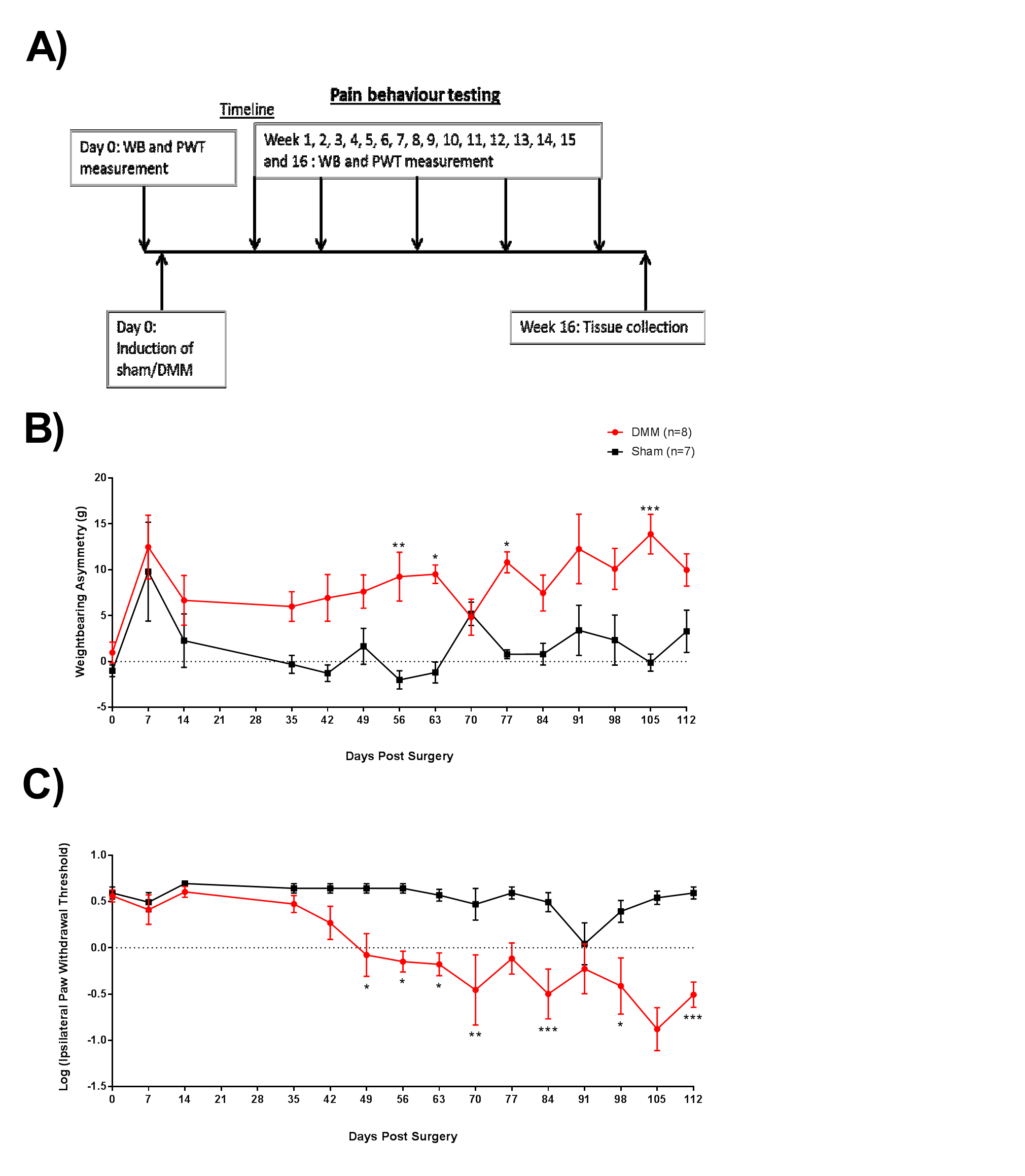


**Figure S1.** **A)** Timeline for the behavioural experiment. Changes in pain behavior in DMM- and sham-treated mice over the 16 week time course in; **B)** weight bearing (WB) difference between ipsilateral and contralateral hindpaws and **C)** hindpaw withdrawal threshold (PWT) to mechanical punctuate stimulation. Statistical analysis comparing WB and PWT between ipsilateral and contralateral hindpaws used two-way ANOVA with Bonferroni’s *post hoc* tests for comparison of sham and DMM. *p<0.05, **p<0.01. Data are expressed as mean ± SEM of differences in WB between ipsilateral and contralateral hindpaws and hindpaw PWTs (n=7, sham; n=8, DMM).


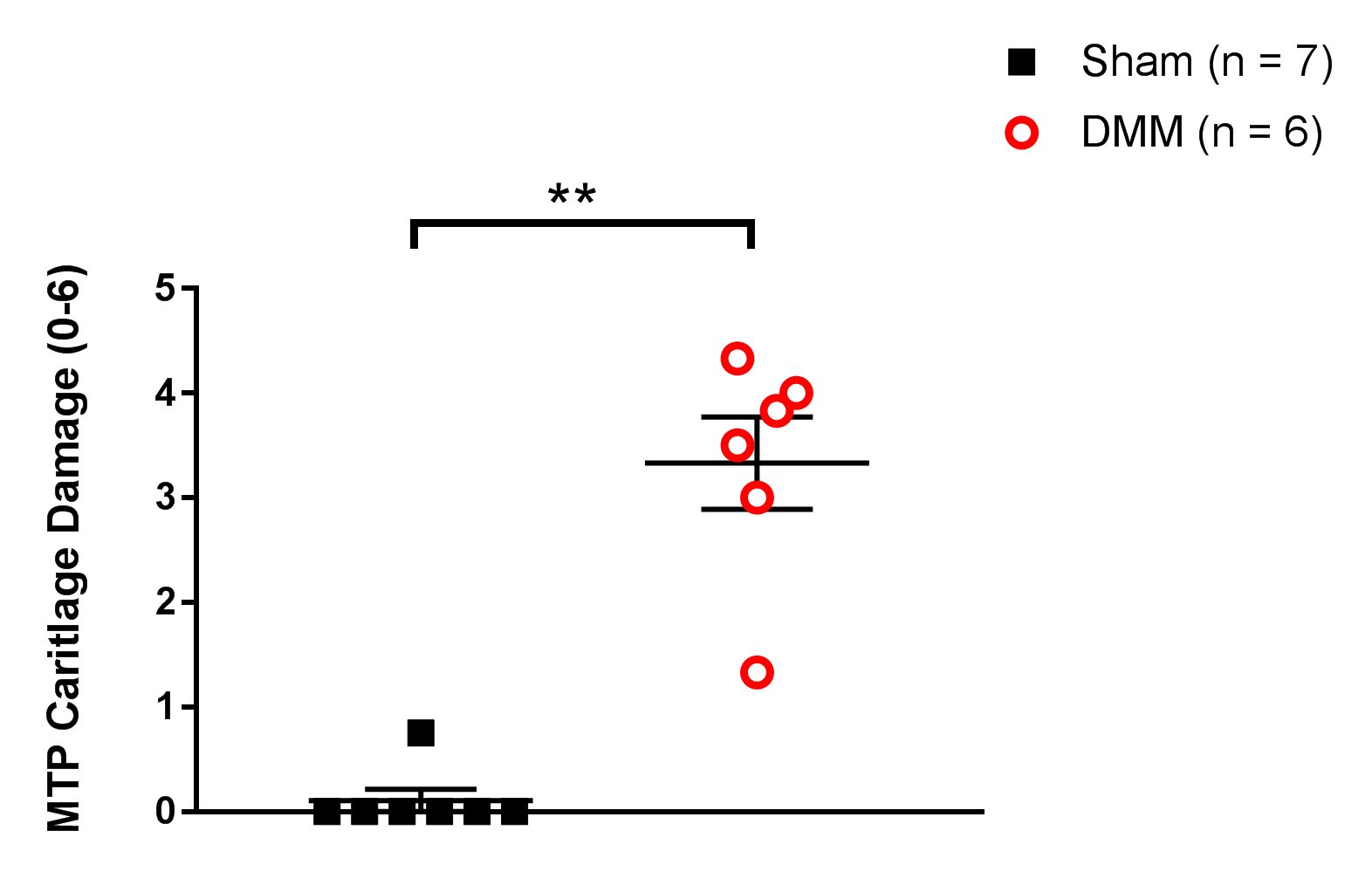


A

C

B

**
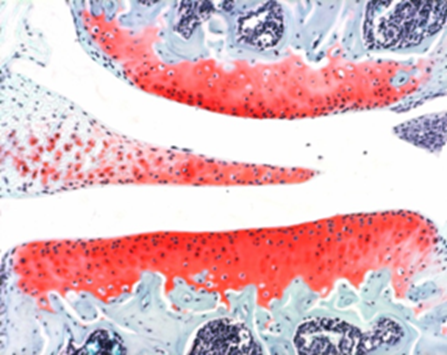
**
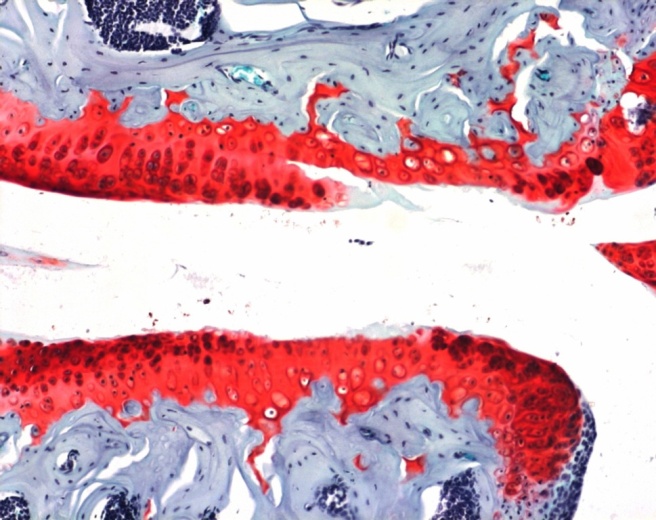


**Figure S2. A)** Cartilage damage quantified in the medial tibial plateau of the ipsilateral knee joint. Representative images of cartilage damage in a sham operated knee joint **(B)** or a DMM operated knee joint **(C)**. Data were analysed by Mann Whitney U test, ** = P<0.01 DMM vs Sham.

| Lipid | Arachidonic acid | | Palmitoleic acid | | Linoleic acid | | LysoPC(18:1) | |
| --- | --- | --- | --- | --- | --- | --- | --- | --- |
|  | Peak area | RT | Peak area | RT | Peak area | RT | Peak area | RT |
| MEAN | 809862.03 | 0.42 | 3653536.92 | 0.42 | 12113364.76 | 0.45 | 2081100.10 | 0.54 |
| SD | 40100.30 | 0.00 | 139557.06 | 0.00 | 379665.71 | 0.00 | 77716.79 | 0.01 |
| RSD (%) | 4.95 | 0.22 | 3.82 | 0.22 | 3.13 | 0.21 | 3.73 | 1.83 |

**Table S1.** The peak areas and retention times (RT) of different lipids observed in pooled QC samples (n=6) prepared from plasma samples of sham and DMM mice. The RSD (%) of peak areas and RT are less than 15% and 2%, respectively, demonstrating the excellent stability and reproducibility of the UHPLC-HR-MS global lipidomics platform.

| Lipid | LysoPC(16:0) | | Oleic Acid | | LysoPC(15:0) | | Stearic acid | | ceramide(d18:1/16:0) | |
| --- | --- | --- | --- | --- | --- | --- | --- | --- | --- | --- |
|  | Peak area | RT | Peak area | RT | Peak area | RT | Peak area | RT | Peak area | RT |
| MEAN | 7653107.10 | 0.52 | 21136005.10 | 0.59 | 48916.03 | 0.67 | 7603136.07 | 0.78 | 12788.33 | 1.31 |
| SD | 339922.96 | 0.00 | 2555015.88 | 0.00 | 3908.31 | 0.01 | 166168.15 | 0.00 | 1987.84 | 0.00 |
| RSD (%) | 4.44 | 0.18 | 12.09 | 0.16 | 7.99 | 1.98 | 2.19 | 0.12 | 15.54 | 0.02 |

**Table S1 (continued).** The peak areas and retention times (RT) of different lipids observed in pooled QC samples (n=6) prepared from plasma samples of sham and DMM mice. The RSD (%) of peak areas and RT are less than 15% and 2%, respectively, demonstrating the excellent stability and reproducibility of the UHPLC-HR-MS global lipidomics platform.


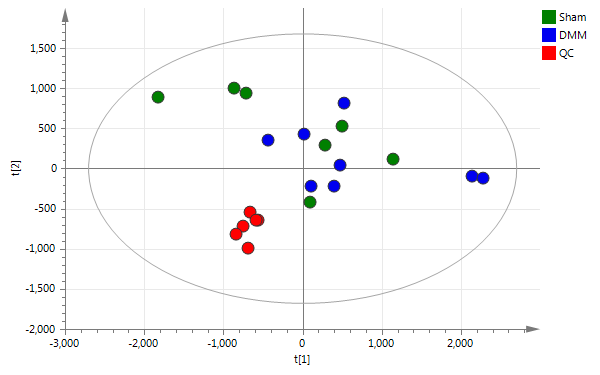


**Figure S3.** PCA score plot of plasma from DMM mice (n = 8, blue), sham mice (n = 7, green) and pooled QCs (n = 6, red). The QCs are clustered well showing our results are valid (R2X = 0.644 and Q2 = 0.488).


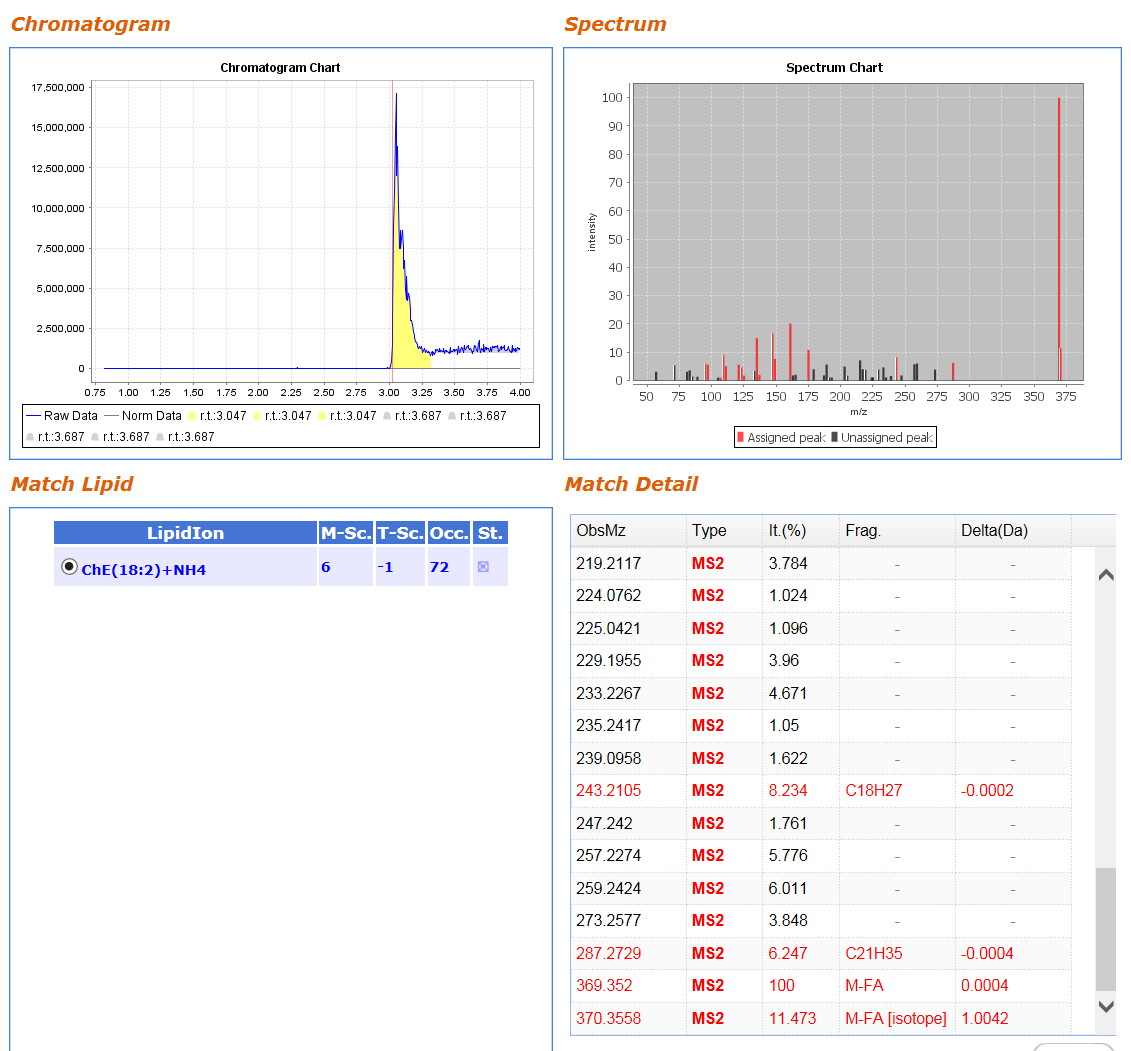


**Figure S4.** MS/MS Spectrum of CE(18:2) as identified by LipidSearch software (Thermo, UK)


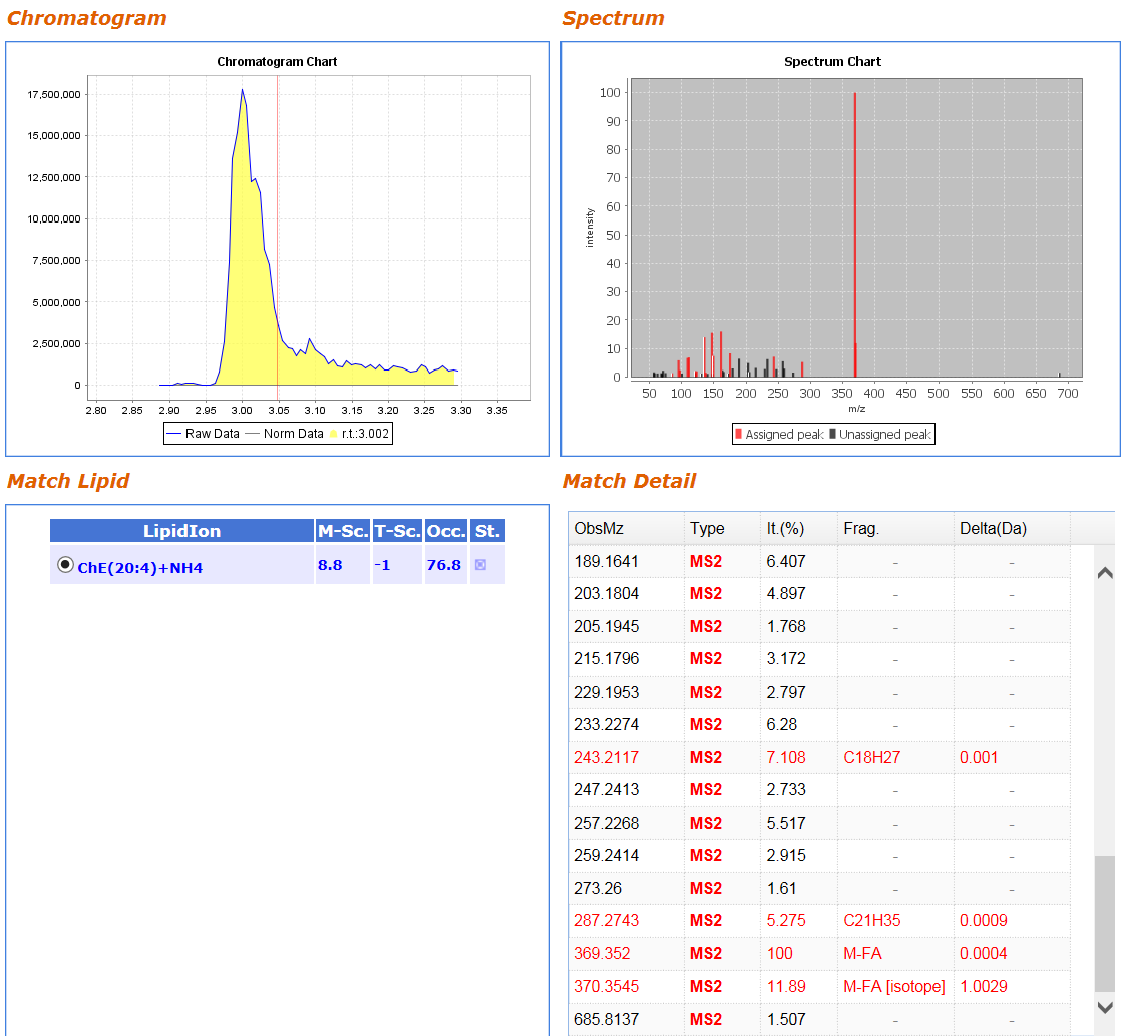


**Figure S5.** MS/MS Spectrum of CE(20:4) as identified by LipidSearch software (Thermo, UK)


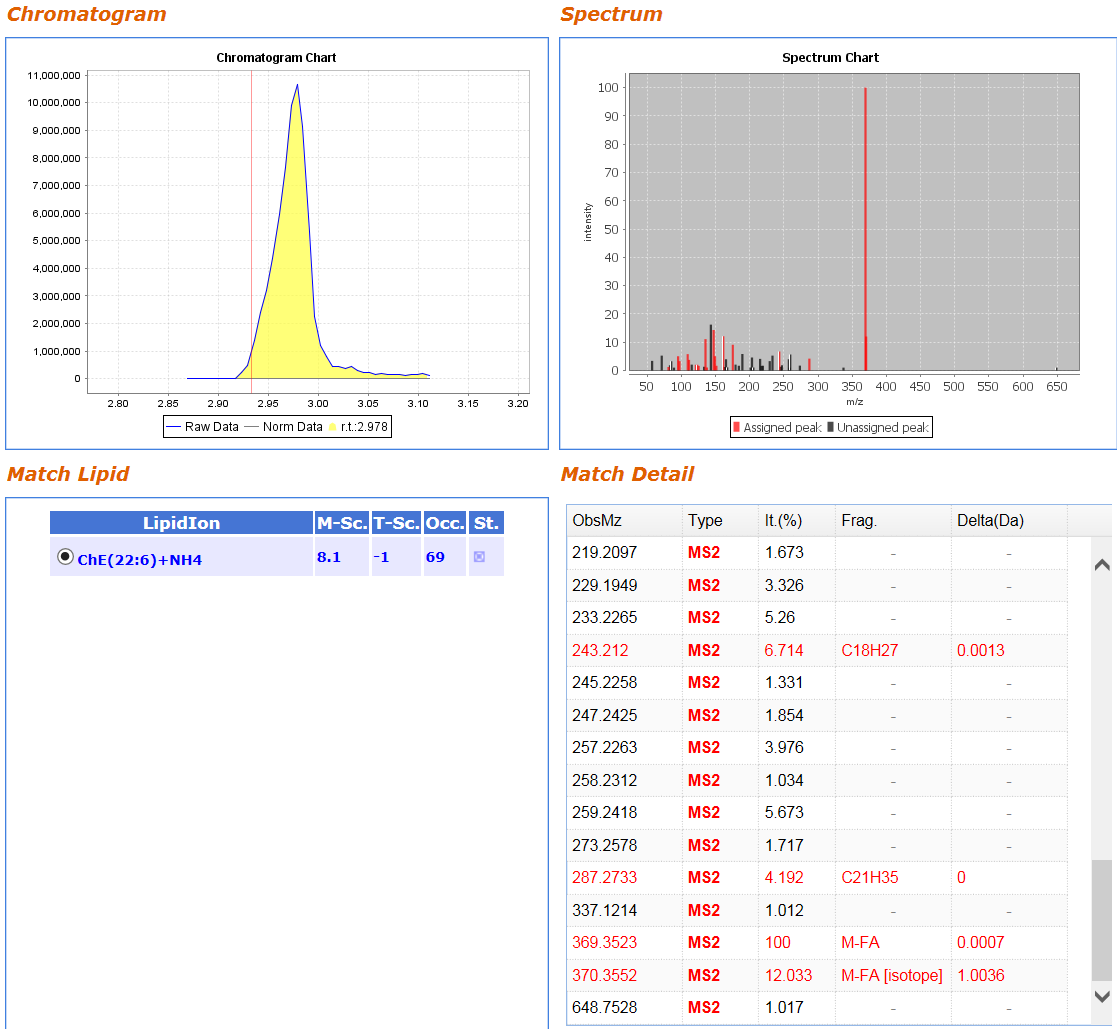


**Figure S6.** MS/MS Spectrum of CE(22:6) as identified by LipidSearch software (Thermo, UK)


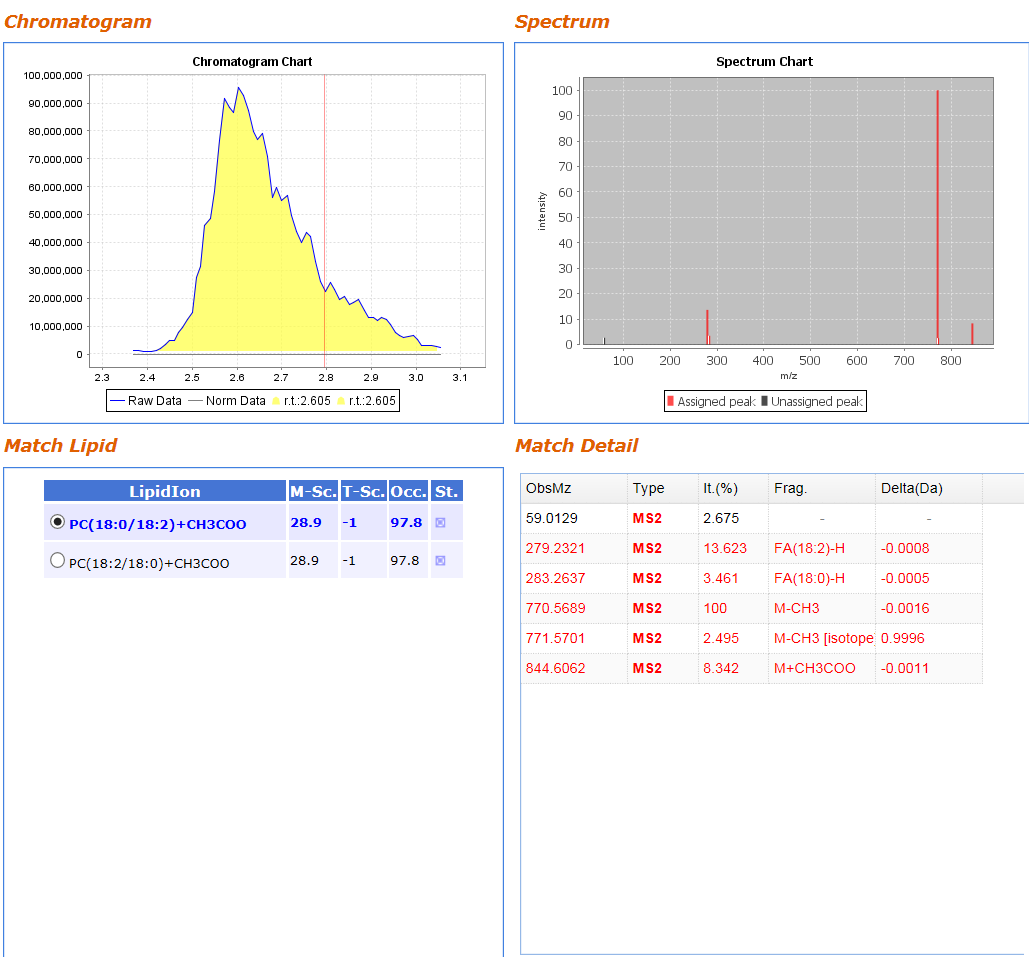


**Figure S7.** MS/MS Spectrum of PC(18:0/18:2) as identified by LipidSearch software (Thermo, UK)


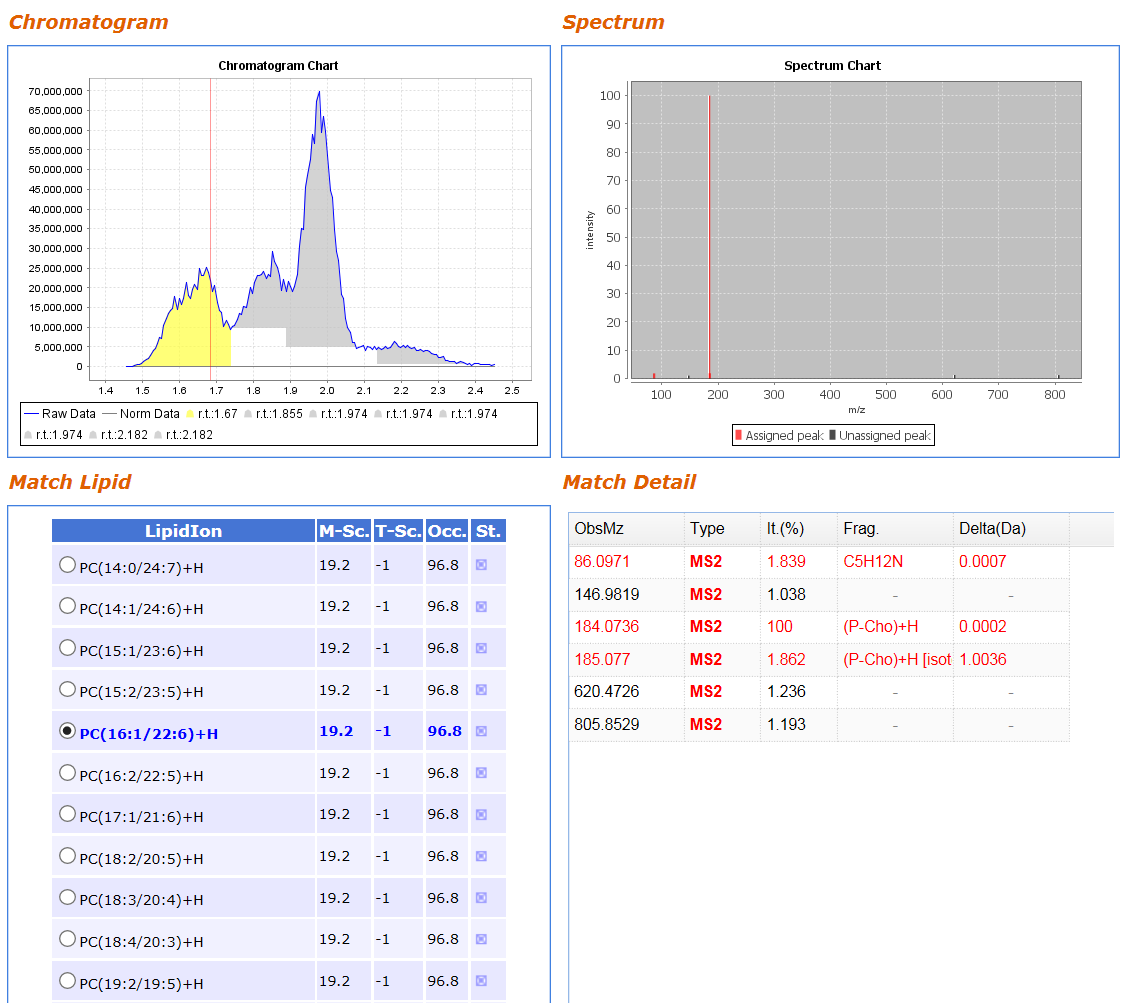


**Figure S8.** MS/MS Spectrum of PC(38:7) as identified by LipidSearch software (Thermo, UK)


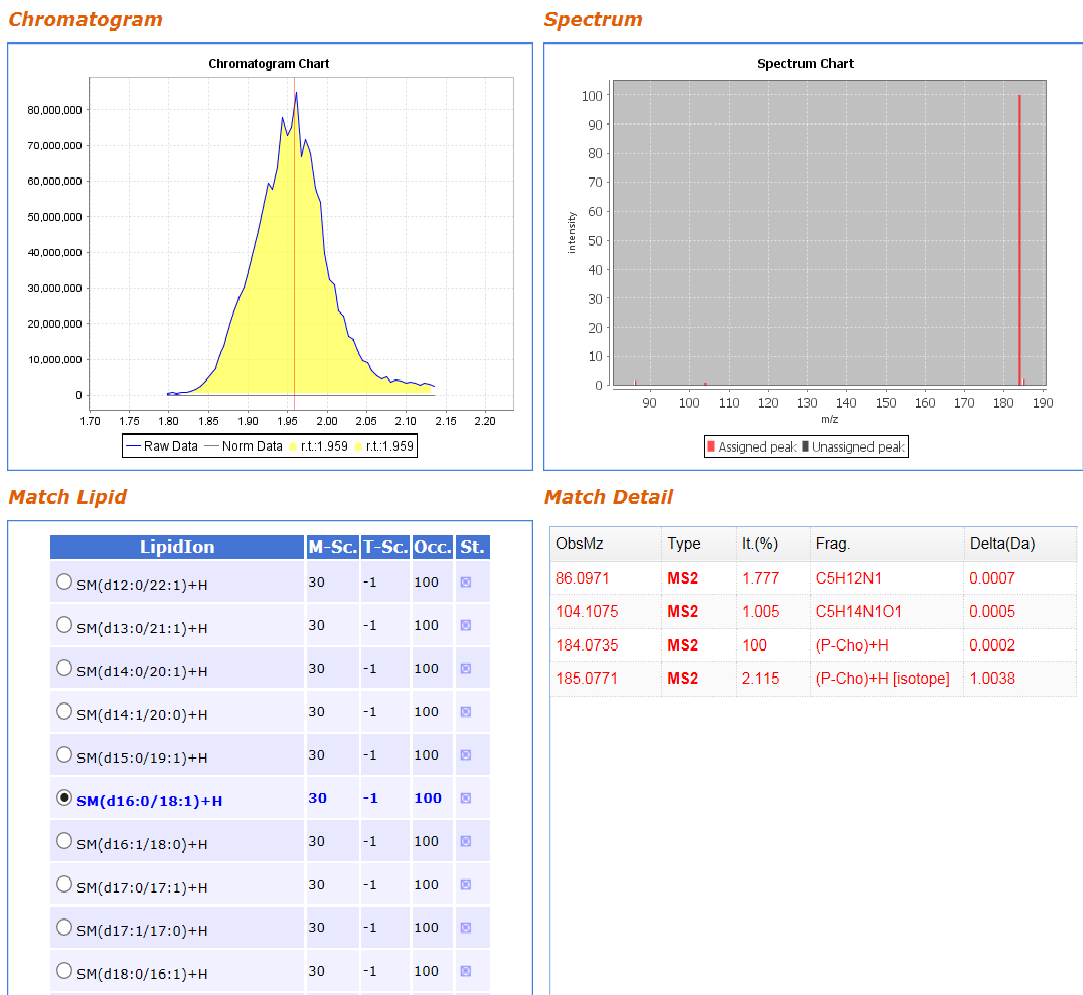


**Figure S9.** MS/MS Spectrum of SM(d34:1) as identified by LipidSearch software (Thermo, UK)

|  | Total compounds | Hits | Raw p | -log(p) | Holm adjust | FDR | Impact |
| --- | --- | --- | --- | --- | --- | --- | --- |
| Steroid biosynthesis | 35 | 1 | 0.006564 | 5.0261 | 0.039385 | 0.019078 | 0.03226 |
| Sphingolipid metabolism | 21 | 1 | 0.006805 | 4.9901 | 0.039385 | 0.019078 | 0.03448 |
| Linoleic acid metabolism | 6 | 1 | 0.019078 | 3.9592 | 0.076313 | 0.019078 | 0.25 |
| alpha-Linolenic acid metabolism | 9 | 1 | 0.019078 | 3.9592 | 0.076313 | 0.019078 | 0.2 |
| Glycerophospholipid metabolism | 30 | 1 | 0.019078 | 3.9592 | 0.076313 | 0.019078 | 0.07895 |
| Arachidonic acid metabolism | 36 | 1 | 0.019078 | 3.9592 | 0.076313 | 0.019078 | 0 |

**Table S2.** Metabolic pathway analysis (MetPA) results as carried out in MetaboAnalyst.

r = 0.4, p = 0.2


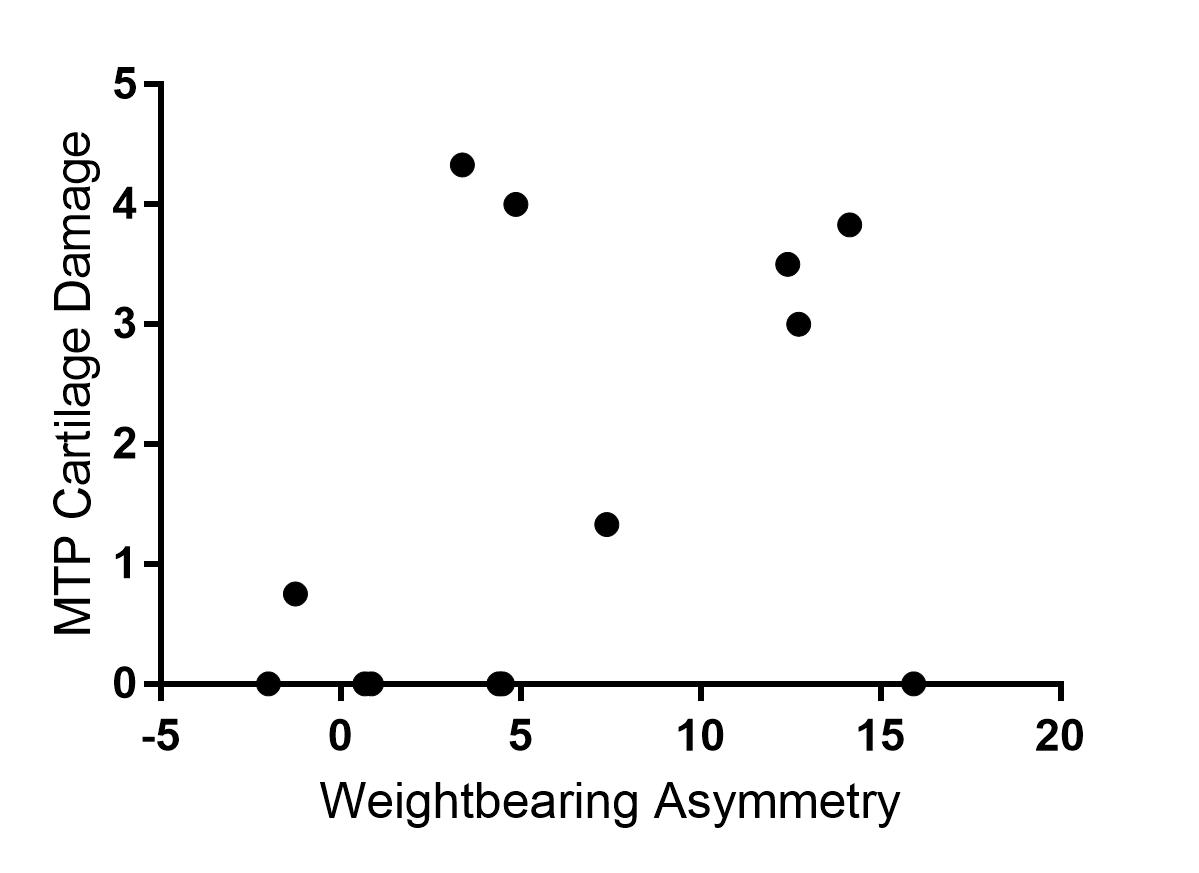


**Figure S10.** There was no significant correlation between weight-bearing asymmetry and cartilage damage. Data analysed by Pearsons Correlation Co-Efficient. P and r values are shown.


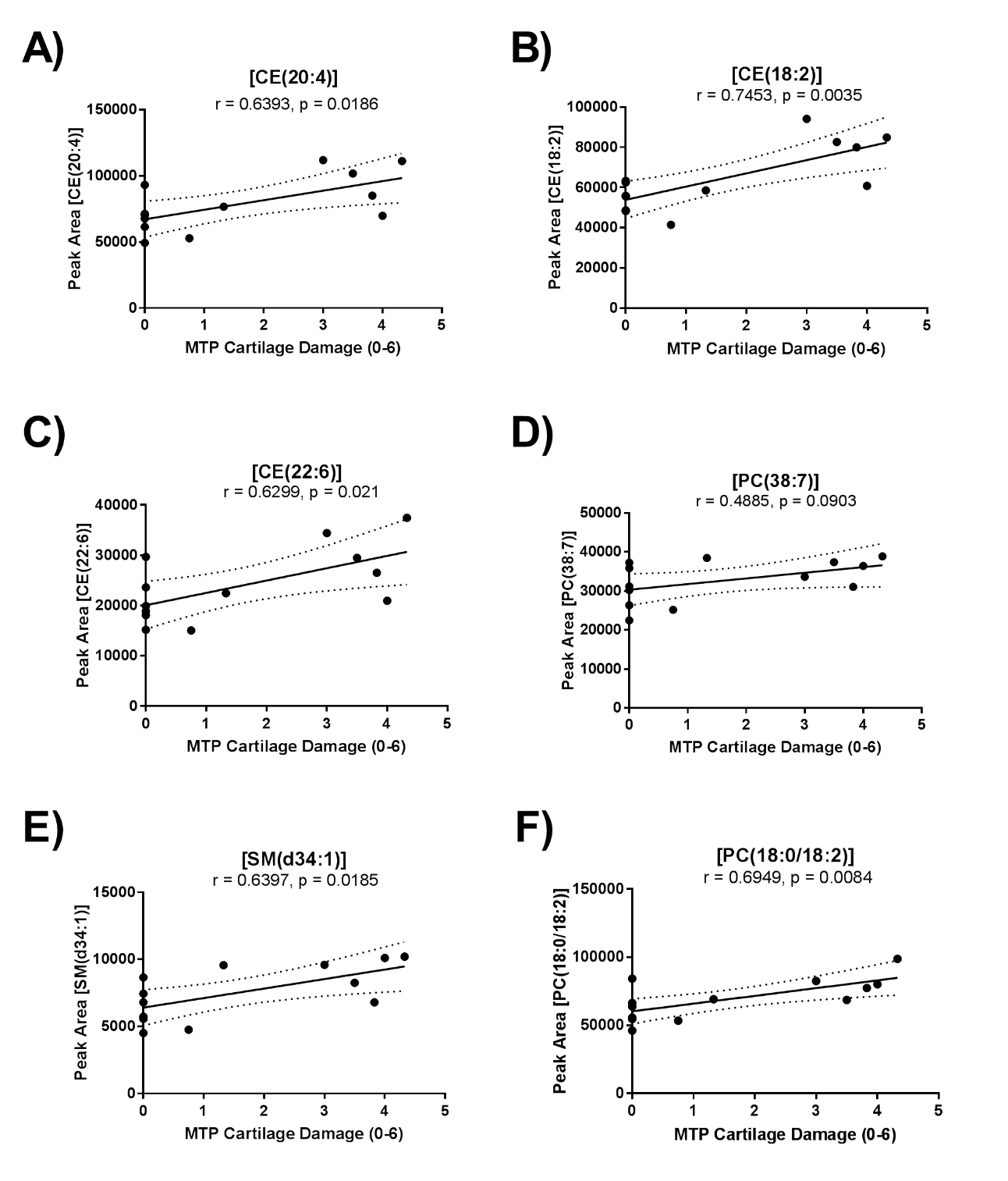


**Figure S11.** Correlation between levels of lipid metabolites and cartilage damage in the medial tibial plateau 16 weeks post DMM/sham surgery. Data analysed by Pearson’s Correlation Co-efficient. A positive correlation for all lipid biomarkers was observed. P and r values are shown.
